# Other cortical dysfunctions during visual and sensitive migraine aura

**DOI:** 10.1186/1129-2377-14-S1-P115

**Published:** 2013-02-21

**Authors:** JZT Zidverc-Trajkovic, IP Petrusic, AP Podgorac, AR Radojicic, NS Sternic

**Affiliations:** 1 Headache Center, Neurology Clinic, Cilinical Center of Serbia, Serbia and Montenegro; 2 School of Medicine, Unversity of Belgrade, Serbia, Serbia and Montenegro; 3 Headache Center, Neurology Clinic, Cilinical Center of Serbia, UK

## 

Migraine aura is the cortical phenomenon for which spreading depression is suggested to be the underlying pathophysiological process. The most common form of migraine aura is visual, followed by the combination of visual and sensitive aura. The majority of these patients also report the presence of the transient disturbances related to other cortical functions (CF) during aura. The aim of this study was to identify disturbances of CF during visual and/or sensitive aura in our group of patients. From the database of patients treated in our Headache Center from 2005 to 2011, 89 patients with migraine with visual and/or sensitive aura were indentified and 60 of them accepted to participate in this study. The questionnaire was filled in by the patient in the presence of the doctor (I.P.). The questionnaire consisted of 17 questions related to color and face recognition; agnosia, memory and speech disturbances, spatial disorientation, apraxia and hallucinations during aura. The demographic data, frequency and duration of aura, as well as the type and number of the CF disturbances were compared between patients with and without CF disturbances. CF disorders were reported by 39 (65.1%) patients. The aura duration was longer in the patients with CF disturbances than without it (28.5 ± 16.4 vs. 19.8 ± 11.2; minutes, p>0.05). The most frequently reported were motor dysphasia (82.1%), dysnomia (30.7%), and impaired recalling .The patients with visual and sensitive aura had longer duration of aura than the patients with only visual aura. The results suggest that during the visual and sensitive aura in patients with migraine disorders of other CF are frequent and related to the duration of the aura.
